# DNA epigenome editing using CRISPR-Cas SunTag-directed DNMT3A

**DOI:** 10.1186/s13059-017-1306-z

**Published:** 2017-09-18

**Authors:** Yung-Hsin Huang, Jianzhong Su, Yong Lei, Lorenzo Brunetti, Michael C. Gundry, Xiaotian Zhang, Mira Jeong, Wei Li, Margaret A. Goodell

**Affiliations:** 1 Department of Developmental Biology, Baylor College of Medicine, Houston, TX 77030 USA; 2 Stem Cells and Regenerative Medicine Center, Baylor College of Medicine, Houston, TX 77030 USA; 3 Center for Cell and Gene Therapy, Baylor College of Medicine, Houston, TX 77030 USA; 4 Division of Biostatistics, Dan L Duncan Cancer Center, Baylor College of Medicine, Houston, TX 77030 USA; 5 Department of Molecular and Human Genetics, Baylor College of Medicine, Houston, TX 77030 USA

## Abstract

**Background:**

DNA methylation has widespread effects on gene expression during development. However, our ability to assign specific function to regions of DNA methylation is limited by the poor correlation between global patterns of DNA methylation and gene expression.

**Results:**

Here, we utilize nuclease-deactivated Cas9 protein fused to repetitive peptide epitopes (SunTag) recruiting multiple copies of antibody-fused de novo DNA methyltransferase 3A (DNMT3A) (dCas9-SunTag-DNMT3A) to amplify the local DNMT3A concentration to methylate genomic sites of interest. We demonstrate that dCas9-SunTag-DNMT3A dramatically increases CpG methylation at the *HOXA5* locus in human embryonic kidney (HEK293T) cells. Furthermore, using a single guide RNA, dCas9-SunTag-DNMT3A is able to methylate a 4.5-kb genomic region and repress *HOXA5* gene expression. Reduced representation bisulfite sequencing and RNA-seq show that dCas9-SunTag-DNMT3A methylates regions of interest with minimal impact on the global DNA methylome and transcriptome.

**Conclusions:**

This effective and precise tool enables site-specific manipulation of DNA methylation and may be used to address the relationship between DNA methylation and gene expression.

**Electronic supplementary material:**

The online version of this article (doi:10.1186/s13059-017-1306-z) contains supplementary material, which is available to authorized users.

## Background

DNA methylation, an epigenetic modification with widespread effects on gene expression, controls tissue specification during development [1, 2]. In vertebrates, DNA methylation is maintained by DNA methyltransferase 1 (DNMT1) [3] and established by DNMT3A and DNMT3B [4]. Cytosine methylation on CpG dinucleotides is the predominant form of methylation in the mammalian genome [5]; however, CpH methylation (H = A or C or T) also is found in embryonic stem cells (ESCs) and adult brain tissue, though not in most somatic cells. High activity of DNMT3A is thought to be largely responsible for CpH methylation [5–8].

Abnormal DNA methylation has been observed in cancer for more than two decades, with many investigations focusing on promoter hypermethylation, which leads to tumor suppressor gene silencing [9, 10]. However, DNA methylation profiling of DNMT3A-mutant acute myeloid leukemia (AML) patient samples showed regional increases as well as decreases in methylation, and changes in gene expression were poorly correlated to changes in DNA methylation [11]. Additionally, many genes with regional hypermethylation are not associated with cancer pathogenesis [12]. These data highlight our limited ability to correctly assign specific functions to DNA methylation in different genic regions.

In order to investigate the relationships between DNA methylation and gene expression, there has been significant interest in generating tools to specifically methylate or demethylate regions in a targeted manner. Previously, researchers were able to demethylate specific regions using either transcription activator-like effector (TALE)-ten-eleven translocation methylcytosine dioxygenase (TET1) or deactivated clustered regularly interspaced short palindromic repeats (CRISPR) associated protein 9 (dCas9)-TET1 fusion protein [13, 14]. Furthermore, researchers have fused dCas9 with the catalytic domain of DNMT3A; however, these tools only methylated up to about 60% at a limited number of CpGs near the guide RNA binding sites [15, 16]. One possible explanation for the limited efficacy of these tools is the requirement for DNMT3A to form tetramers in order to methylate DNA efficiently.

## Results

**Fig. 1:**
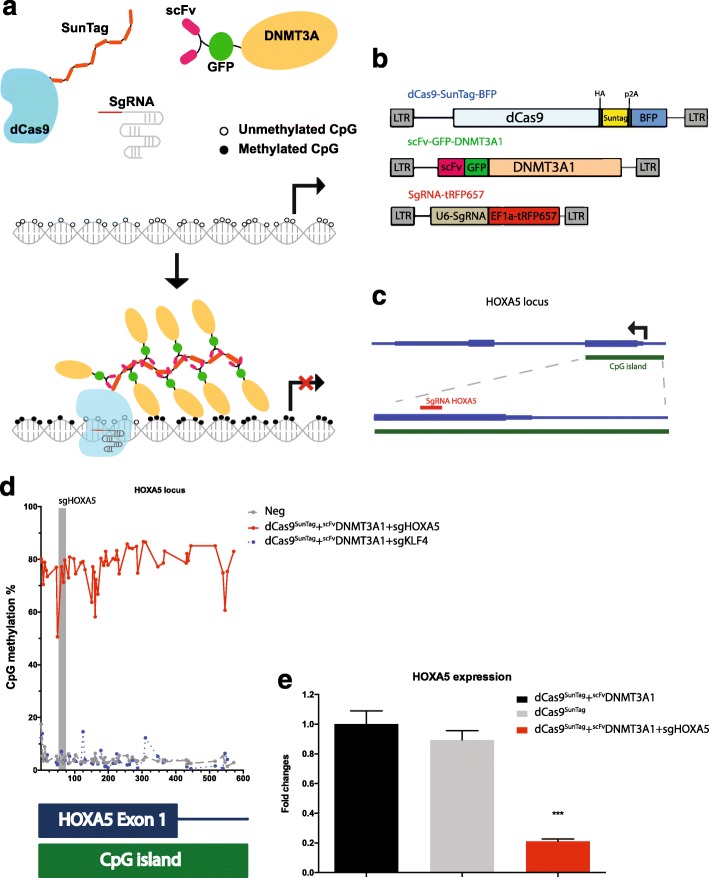
dCas9-SunTag-DNMT3A methylates the *HOXA5* promoter and suppresses gene expression. **a** Schematic of dCas9-SunTag-DNMT3A. Deactivated Cas9 (*dCas9*) was fused to SunTag epitopes and the single-chain variable fragment (*scFv*) was fused to green fluorescent protein (*GFP*) and DNMT3A1. With guide RNAs, multiple copies of ^scFv^DNMT3A1 can be directed to specific loci and methylated regions of interest. **b** Lentiviral vectors of dCas9-SunTag-DNMT3A. The dCas9^SunTag^ vector contains the p2A self-cleaving peptide to separate dCas9^SunTag^ and blue fluorescent protein (*BFP*). Guide RNA vector contains the RFP657 fluorescence marker. **c** The guide RNA design at the *HOXA5* locus. Guide RNA HOXA5 (*sgHOXA5*) was located in exon 1 of *HOXA5* gene. **d** The percent of methylation across individual CpGs as determined by deep sequencing of amplicons across the locus. **e** The fold change relative to dCas9^SunTag^ + ^scFv^DNMT3A1 as analyzed by quantitative real-time PCR for HOXA5 expression, measured 30 days after transduction of indicated constructs. ***P < 0.001 by Student *t* test

Here, we have focused on establishing a system for targeted DNA methylation that can function over large regions such as an entire CpG island or a DNA methylation canyon [17]. Recent advances in CRISPR-Cas9 technology have allowed efficient targeting of proteins. In order to methylate DNA efficiently, we employed the SunTag system [18, 19], which is an amplifier based on multiple antibody epitopes recognized by a single-chain variable fragment (scFv). dCas9 is fused to the epitopes, and the scFv is fused to the protein of interest, which in our case is DNMT3A (Fig. 1a). We hypothesized that co-expression of the two components along with guide RNA would result in robust DNA methylation.

For our studies, we utilized three lentiviral vectors carrying the main components (Fig. 1b). First, dCas9 was linked to the SunTag along with blue fluorescent protein (BFP, separated by a p2A sequence) for tracking expression. Second, DNMT3A was fused at the N-terminus to scFv and superfolder green fluorescent protein (GFP). We initially generated three versions of DNMT3A: the long isoform (DNMT3A1), the short isoform (DNMT3A2), and the catalytic domain alone (DNMT3Ame) (Additional file 1: Figure S1a). Finally, the guide RNA was expressed along with far-red fluorescent protein for tracking purposes.

To test this system, we chose a large CpG island that straddles both a promoter and the first exon of the *HOXA5* locus (Fig. 1c). This CpG island is essentially unmethylated in most cell types with variable gene expression depending on the cell type, and has been implicated in AML [20], thus offering a test locus for our system. Guide RNA HOXA5 was designed to bring the proteins to the first exon of the locus. We first examined the efficacy of the dCas9-SunTag-DNMT3A system after transient transfection of the constructs into human embryonic kidney (HEK293T) cells. Forty-eight hours after transfection, fluorescence-positive cells were sorted (Additional file 1: Figure S2), and DNA and RNA were extracted for bisulfite sequencing and quantitative PCR (qPCR), respectively.

Untreated negative control samples (hereafter referred to as “neg”) showed that baseline methylation at the *HOXA5* locus was around 2–4% in HEK293T cells (Additional file 1: Figure S1b). A 2–5% increase in methylation at a subset of CpGs and a 20% *HOXA5* mRNA repression were seen when ^scFv^DNMT3A1 was specifically targeted to the *HOXA5* locus by dCas9^SunTag^ and guide RNA HOXA5 (Additional file 1: Figure S1c,d), suggesting that the dCas9-SunTag-DNMT3A system was modestly effective in this context. Notably, the nonproportional repression of transcription is a result of both dCas9 binding and a slight increase of DNA methylation (Additional file 1: Figure S3). The methylation level also increased by 2–5% in the sample with ^scFv^DNMT3A2 (along with the guides and dCas9^SunTag^), but no significant gene expression changes were observed using this construct (Additional file 1: Figure S1e,f). Similarly, no significant methylation or gene expression changes were observed when the ^scFv^DNMT3Ame construct was targeted to the locus (Additional file 1: Figure S1g, h). In addition, no methylation changes at the *HOXA5* locus were observed when cells globally expressed dCas9^SunTag^ and ^scFv^DNMT3A constructs without adding guide RNAs (Additional file 1: Figure S1b). Together, these data suggested that the dCas9-SunTag-DNMT3A system offered some targeted DNA methylation, but the efficacy was limited, perhaps due to limited exposure time when the vectors were transiently expressed.

We next compared the efficiency of our system directly to other available DNA methylation tools, specifically dCas9-DNMT3A [21] and dCas9-DNMT3ACD [15]. Although each of these tools requires a different time frame, we conducted uniform experiments with transient transfection and harvested cells after 72 h. We used the three systems to target the *BACH2*, *HOXA5*, and *KLF4* loci. We chose *BACH2*, as dCas9-DNMT3ACD and guide RNA BACH2 have been shown to methylate the locus [15]. Fluorescence-positive cells were sorted, and then DNA was extracted for bisulfite sequencing. We conducted these experiments with three biological replicates. First, we examined specificity by introducing the three systems without adding guide RNAs. We found that dCas9-DNMT3ACD conveyed strong off-target DNA methylation across CpGs in all three loci (Additional file 1: Figure S4a,c,e), while dCas9-DNMT3A and our dCas9-SunTag-DNMT3A system showed minimal off-target effects. When guide RNAs were added, dCas9-DNMT3ACD also showed the most significant increase in methylation (about 40–80%). Our dCas9-SunTag-DNMT3A system increased methylation by 10–30% at the three loci, whereas dCas9-DNMT3A increased methylation by 10% at the *HOXA5* locus only and not at the other two loci (Additional file 1: Figure S4b,d,f). Notably, we analyzed expression levels of the components using flow cytometry (Additional file 1: Figure S5). As Additional file 1: Figure S5d shows, dCas9-SunTag-DNMT3A had higher GFP expression and less off-target methylation than dCas9-DNMT3ACD (Additional file 1: Figure S4), suggesting that higher expression levels do not necessarily increase off-target methylation. While these results suggest that dCas9-DNMT3ACD had the strongest methylation efficiency, they also demonstrated loss of specificity. Additionally, the dCas9-DNMT3A system was only able to methylate one of the three loci tested. Our dCas9-SunTag-DNMT3A system increased methylation at all three of the loci without significant off-target methylation, which suggests that it may be an effective tool in at least the contexts tested here.

To improve the efficiency of our system, we generated stably expressing cells by using lentiviral transduction of the constructs and focused on expression of DNMT3A1. HEK293T cells were transduced with lentiviral vectors harboring dCas9^SunTag^, ^scFv^DNMT3A1, and guide RNAs targeting the *HOXA5* locus. After 48 h, fluorescence-positive cells were purified by flow cytometry and cultured. Thirty days later, fluorescence-positive cells were repurified for analysis of DNA methylation and gene expression.

DNA methylation was analyzed by deep sequencing of amplicons across the *HOXA5* locus after bisulfite treatment of the genomic DNA. The low steady-state level of DNA methylation (2–5%) across this CpG island was verified by the non-transduced negative control (Additional file 1: Figure S6). When DNMT3A1 alone was constitutively over-expressed, DNA methylation was very high (Additional file 1: Figure S6). Remarkably, however, cells that also contained the SunTag construct in the absence of guide RNA exhibited low levels of DNA methylation, similar to those with dCas9 alone (Additional file 1: Figure S6). These data suggest that the dCas9^SunTag^ component binds the free ^scFv^DNMT3A1, limiting spurious DNA methylation even when there are no guide RNAs present.

We then examined the methylation impact in the presence of guide RNAs. When an irrelevant guide RNA against the *KLF4* locus was included along with dCas9^SunTag^ and ^scFv^DNMT3A1 constructs, we observed low methylation across the locus (Fig. 1d). However, when the construct containing the *HOXA5* guide RNA was included, methylation across the entire locus was around 80% (Fig. 1d). These data indicate efficient targeted DNA methylation using the dCas9-SunTag-DNMT3A constructs and guide RNAs. Together, these data show that DNMT3A is a potent methylator that can be effectively harnessed with a combination of the SunTag system and dCas9 targeting system. When guides are present, the DNA methylation activity is specifically directed (Fig. 1d).

In order to examine the impact of targeted DNA methylation on gene expression, we performed qPCR on the transduced samples. We observed that targeting of ^scFv^DNMT3A1 to the *HOXA5* locus by dCas9^SunTag^ and guide RNA resulted in 80% repression of *HOXA5* gene expression (Fig. 1e). These results indicate that the dCas9-SunTag-DNMT3A system can not only methylate CpGs in specific regions but can also repress gene expression.

**Fig. 2:**
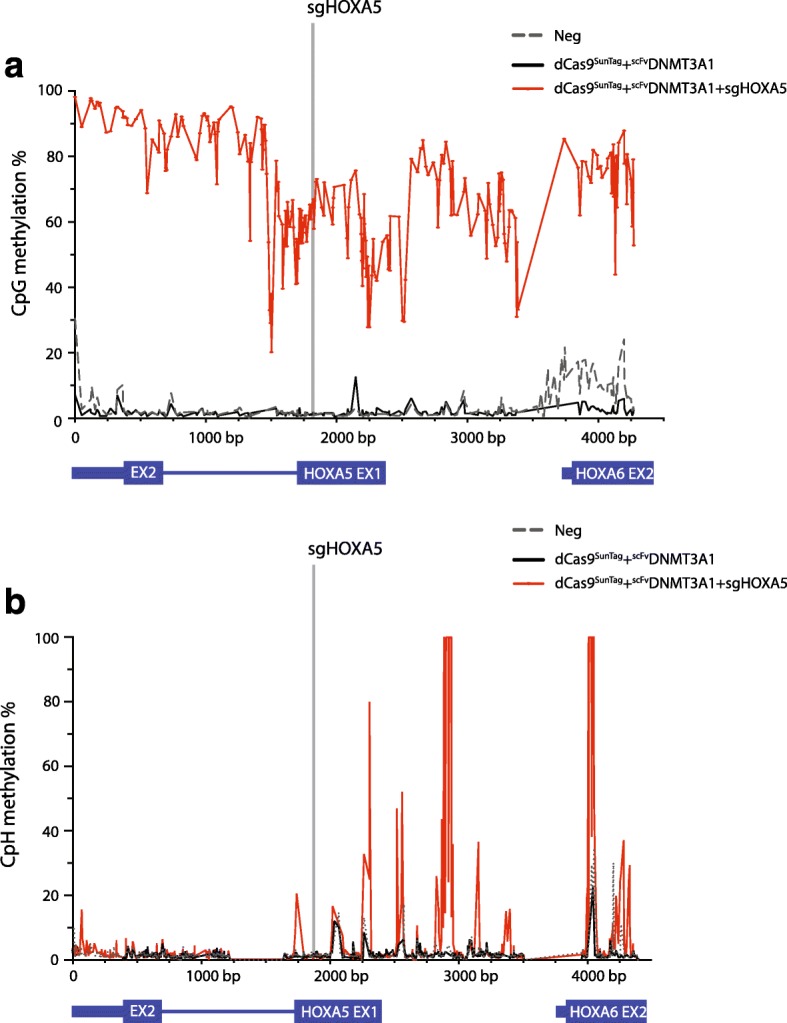
dCas9-SunTag-DNMT3A methylates CpGs and CpHs within 4.5 kb of *HOXA5* locus. **a** The percent of methylation across individual CpGs as determined by deep sequencing of amplicons within a 4.5-kb window of *HOXA5* locus. **b** The percent of methylation across individual CpHs as determined by deep sequencing of amplicons within a 4.5-kb window of *HOXA5* locus

De novo DNA methylation is thought to become highly effective when many DNMT3 molecules are recruited to a site [22]. Because the local concentration of DNMT3A could be quite high with the SunTag system due to the amplification effect of the repetitive scFv binding sites, we considered the possibility that DNA methylation would extend further than the 600 bp initially observed. To examine this, we performed deep sequencing of multiple bisulfite PCR amplicons around the target regions. We observed that the dCas9-SunTag-DNMT3A system was able to methylate all CpGs detected in a 4.5-kb window of the *HOXA5* gene and part of the adjacent *HOXA6* gene (Fig. 2a). Surprisingly, we also observed that the dCas9-SunTag-DNMT3A system methylated some CpHs (H = A or C or T) in the same *HOXA5* and *HOXA6* window. Previous reports demonstrated that methylated CpA (mCpA) is the most abundant form of CpH methylation, followed by mCpT and mCpC [7, 23]. We thus wondered whether ^scFv^DNMT3A1 guided to *HOXA5* loci likewise increased mCpA more dramatically than mCpT and mCpC. As expected, mCpA was the dominant form of CpH methylation at the *HOXA5* loci; mCpC was the least dominant form (Additional file 1: Figure S7). Using knock-down and knock-out experiments, previous literature has suggested that DNMT3A is necessary for CpH methylation [7, 8]; our data support the concept that concentrated expression of DNMT3A will induce CpH methylation.

In order to further validate the method and examine the effects of DNA methylation changes on gene expression, we chose to examine a different locus, the *KLF4* gene. This locus harbors a DNA methylation canyon [17] (Additional file 1: Figure S8a) that shows a sharp transition from very low methylation to high methylation. While unmethylated in most normal tissues according to The Cancer Genome Atlas (TCGA) data, this transition region becomes highly methylated in many tumor types (Additional file 1: Figure S8b). We designed a guide RNA targeting intron 1 (sgKLF4) adjacent to the methylation transition site. Thirty days after lentiviral transduction of HEK293T cells, fluorescence-positive cells were purified, and methylation and gene expression analyses were conducted.

**Fig. 3:**
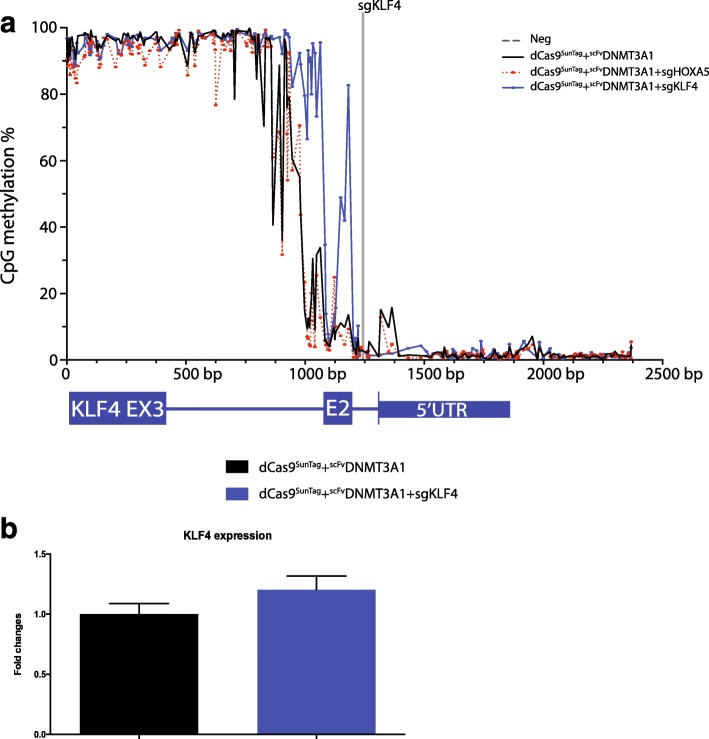
dCas9-SunTag-DNMT3A methylates the intragenic regions of *KLF4* without affecting gene expression. **a** The percent of methylation across individual CpGs as determined using deep sequencing of amplicons in a 2.5-kb window of the *KLF4* locus. The *gray bar* represents the guide RNA binding site. **b** The fold change relative to dCas9^SunTag^ + ^scFv^DNMT3A1 as measured by quantitative PCR for KLF4 expression

In the negative control, exon 2 and the regions upstream exhibited low levels of methylation, while the regions downstream were highly methylated (Fig. 3a). In cells transduced with the dCas9^SunTag^ and ^scFv^DNMT3A1 constructs without the guide RNAs, or with guides targeted to the *HOXA5* locus, there were no observed changes to CpG methylation. However, in the presence of the guide RNAs targeted to the *KLF4* locus, dramatic CpG methylation was seen in close proximity to the target sites (Fig. 3a). Examining the impact of the DNA methylation on gene expression at the locus showed that *KLF4* gene expression changed only slightly in the presence of guide RNAs KLF4, dCas9^SunTag^, and ^scFv^DNMT3A1 constructs (Fig. 3b).

Since we observed long-range methylation at the *HOXA5* locus, we considered whether this effect was due to long-term expression. To examine this, we compared the dCas9-DNMT3A and dCas9-SunTag-DNMT3A systems in the context of long-term expression. We transduced lentiviral particles of dCas9-DNMT3A and guide RNA targeting *HOXA5* and *KLF4* loci on HEK293T cells. After 30 days of incubation, we purified blue fluorescence-positive cells and conducted methylation analysis. We observed around a 20–40% increase of methylation across multiple CpGs at the 4-kb window of the *HOXA5* locus but not at the 2-kb window of the *KLF4* locus (Additional file 1: Figure S9). However, our dCas9-SunTag-DNMT3A conveyed higher methylation efficiency (60–95%) (Figs. 2 and 3) at the *HOXA5* and *KLF4* loci compared to the dCas9-DNMT3A system, potentially because of increased local concentration of DNMT3A and increased chances of multimerization of DNMT3 molecules. Furthermore, from the time point of 72 h (Additional file 1: Figure S4), we found that the *HOXA5* locus was the most sensitive to targeted DNMT3A methylation of the three loci we tested. Therefore, the increased range of methylation we saw at *HOXA5* could be a result of several effects, including long-term expression, sensitivity of the specific region, increased chances of multimerization of DNMT3A molecules, and unknown factors such as chromatin configuration.

**Fig. 4:**
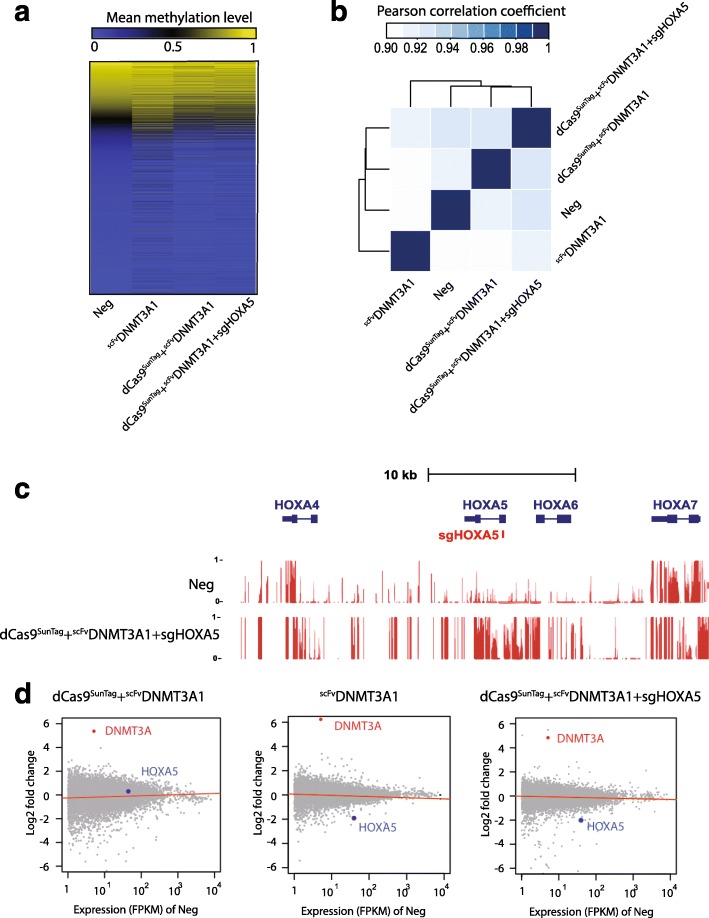
dCas9-SunTag-DNMT3A is capable of methylating 4.5-kb regions of *HOXA5* locus with minimal effects on global methylome and transcriptome. **a** The methylation level of detected CpGs across the genome as determined by reduced representation bisulfite sequencing (RRBS). **b** The correlation between dCas9-SunTag-DNMT3A1-treated samples by Pearson’s correlation method. **c** The methylation status of detected CpGs at *HOXA5* locus by whole genome bisulfite sequencing. The proportional methylation at each analyzed CpG is plotted from 0 to 1 (representing from 0 to 100% methylation). **d** The genome-wide gene expression analysis (log2 fold changes of FPKM) of dCas9-SunTag-DNMT3A1-treated samples compared to non-transduced cells (*Neg*) using RNA-seq. *Red dot* represents relative DNMT3A expression, and *blue dot* represents relative HOXA5 expression compared to Neg cells

When guide RNAs were present along with the dCas9^SunTag^ and ^scFv^DNMT3A constructs, DNA methylation appeared targeted specifically to the vicinity of the guide RNAs, such that the *HOXA5* locus became methylated in the presence of HOXA5 guides, and the *KLF4* locus became methylated in the presence of KLF4 guides. However, we considered whether our construct altered global methylation. To test this, we used reduced representation bisulfite sequencing (RRBS), capturing around 2.6 million CpGs in treated samples. Compared to the untreated negative control sample, cells transduced with the dCas9^SunTag^ and ^scFv^DNMT3A1 constructs without guide RNAs did not show significant changes in DNA methylation. This result suggests that even when not targeted to DNA, the inactive dCas9^SunTag^ likely recruits ^scFv^DNMT3A1; if the dCas9^SunTag^ is not brought to DNA because guides are absent, background activity of the protein complex appears minimal. Even without the dCas9^SunTag^, only about 5% of detected CpGs showed increased methylation in cells transduced with ^scFv^DNMT3A1 alone. Importantly, only regions adjacent to *HOXA5* loci were methylated in the presence of the HOXA5 guide RNAs, suggesting that the dCas9-SunTag-DNMT3A system was site-specific and did not affect genome-wide methylation (Fig. 4a; Additional file 1: Figure S10). When we compared the samples genome-wide by Pearson’s correlation, we observed that the untreated negative control samples clustered closely with both dCas9^SunTag^ and ^scFv^DNMT3A1 constructs with and without HOXA5 guide RNAs, supporting our conclusion that this system has minimal off-target effects (Fig. 4b). Quantitative analysis also showed that methylation increased in 3.12% (187/5982) of detected CpG islands (CGIs) under the constitutive DNMT3A1 over-expression, while only 0.48% and 0.58% of CGIs were hypermethylated under untargeted and targeted dCas9^SunTag^ + ^scFv^DNMT3A1 (Additional file 1: Figure S11), respectively, supporting our observation that dCas9-SunTag-DNMT3A has minimal effects on the global methylome. Notably, using the University of California, Santa Cruz (UCSC) Genome Browser tracks generated from whole-genome bisulfite sequencing, we observed CpG methylation in at least a 10-kb region of *HOXA5* and *HOXA6*, confirming the observations described above (Figs. 2a and 4c).

To test the effect of the dCas9-SunTag-DNMT3A system on global transcriptional status, we conducted RNA-seq on transduced samples and compared their transcriptional status with negative controls. We observed decreased HOXA5 expression in the presence of HOXA5 guide RNA along with the dCas9^SunTag^ and ^scFv^DNMT3A1 constructs, while there were minimal effects on the global transcriptome in the same samples (Fig. 4d). These data support our previous qPCR results (Fig. 1e) and suggest that the dCas9-SunTag-DNMT3A system can modulate specific gene expression with minimal effects on the global transcriptome. In the presence of ^scFv^DNMT3A1 alone, we observed a slight overall decrease of expression in global transcription. The RNA-seq data from all samples also revealed that cells transduced with DNMT3A-bearing constructs had between 30-fold to 80-fold more DNMT3A than did negative controls.

## Discussion

In this report, we engineered dCas9 with the SunTag system to guide DNMT3A1 to specific sites in the genome in order to target DNA methylation in human cells. Using the *HOXA5* locus as an example, we showed that the dCas9-SunTag-DNMT3A system was not only able to methylate CpGs but also CpHs in a 4.5-kb window. These findings indicate that DNMT3A is sufficient for both CpG and CpH methylation in human cells and validate a novel tool to study the function of DNA methylation.

Previous reports utilizing dCas9 and the catalytic domain of DNMT3A combined with multiple guide RNAs showed only up to 60% methylation increases within windows no greater than 200 bp [15, 16]. In addition, targeting of a dCas9-DNMT3A fusion protein increased levels of methylation at a limited number of CpGs [21]. In direct comparison experiments of three available systems (dCas9-DNMT3ACD [15], dCas9-DNMT3A [21], and dCas9-SunTag-DNMT3A) in the context of transient transfection, we showed that dCas9-DNMT3ACD conveyed the highest methylation efficiency but with significant off-target methylation. While the dCas9-SunTag-DNMT3A system showed modest targeted methylation at three tested loci, the dCas9-DNMT3A system only methylated one of three tested loci (Additional file 1: Figure S4). Furthermore, in the context of long-term incubation, the dCas9-SunTag-DNMT3A system also conveyed higher methylation efficiency compared to the dCas9-DNMT3A system (Additional file 1: Figure S9). Of note, an additional dCas9-based DNA methylation system based on a Dnmt3a-Dnmt3L fusion has been described [24]. This system also offers the possibility of increased methylation efficiency due to a multimerization effect and is reported to have reduced off-target events. Ultimately, different tools may be of value in different contexts, and thus independent comparison of several constructs for any given target may be of use. Nevertheless, our data indicate that the tethering of DNMT3A using the SunTag system offers high methylation efficiency with minimal off-target effects.

In our studies, different methylation patterns were observed in the *HOXA5* and *KLF4* loci. We reasoned that this could potentially be due to the location of guide RNAs and the differential sensitivity of different regions to DNA methylation due to unknown factors such as chromatin configuration. For instance, guide RNA HOXA5 was targeted to the center of a large unmethylated region at the *HOXA5* locus (Fig. 4d), and this locus is sensitive to DNMT3A-mediated methylation demonstrated by Additional file 1: Figure S4c,d and Figure S9a. In contrast, the guide RNA for the *KLF4* locus was targeted to the edge of the gene body, which limited the apparent expansion of CpG methylation.

## Conclusions

As DNA methylation changes often occur during tumorigenesis and development in large regions [17, 25, 26], our dCas9-SunTag-DNMT3A may offer an attractive choice to induce DNA methylation changes. In summary, the dCas9-SunTag-DNMT3A DNA methylation system is an effective and accurate tool that allows us to methylate regions of interest with minimal impact on genome-wide methylation. This tool should facilitate our understanding of the role of DNA methylation in different genomic regions in development and cancer.

## Methods

### Vector construction

The dCas9^SunTag^ vector, scFv-sfGFP-VP64 vector, and sgRNA-RFP657 vector were purchased from Addgene (catalog nos. 60903, 60904, and 57824). The scFv-sfGFP-VP64 vector was digested by *Rsr*II and *Spe*I in a 37 °C incubator overnight and gel extracted using a Qiagen gel extraction kit. DNMT3A1, DNMT3A2, and DNMT3A catalytic domains (DNMT3Ame) were cloned from complementary DNA (cDNA) using primers with *Rsr*II and *Spe*I restriction recognition sites (Additional file 2: Table S1). The digested and gel extracted scFv-sfGFP-VP64 vector was then ligated with DNMT3A1, DNMT3A2, and DNMT3Ame at 16 °C overnight. Colonies of three isoforms of scFv-sfGFP-DNMT3A vectors were collected and the sequencing verified.

### Cell culture and lentiviral particle production

The HEK293T cells were incubated in Dulbecco’s modified Eagle’s medium (DMEM) with 10% fetal bovine serum (FBS) and 1% penicillin/streptomycin (P/S) at 5% CO_2_ and 37 °C incubation. The HEK293T cells were transfected with dCas9-SunTag-DNMT3A and dCas9-DNMT3A vectors using Lipofectamine 2000 (Life Technologies) and co-transfected with pMD2.G and psPAX2. Lentiviral particles were collected at 48 h and 72 h after transfection. 4X polyethylene glycol (PEG) (32% PEG6000, 0.4 M NaCl, and 0.04 M 4-(2-hydroxyethyl)-1-piperazineethanesulfonic acid (HEPES)) was added to precipitate viral particles at 4 °C overnight. Viral particles were then centrifuged in 1500 g for 45 min and resuspended in X-VIVO 15 medium (Lonza, Basel, Switzerland).

### Direct comparison

The dCas9-DNMT3A and dCas9-DNMT3ACD vectors were purchased from Addgene (catalog nos. 84569 and 71666). Sequences of guide RNAs targeting *BACH2*, *HOXA5*, and *KLF4* are listed in Additional file 2: Table S1. Three biological replicates were conducted, and HEK293T cells were transfected with all three systems. Seventy-two hours after transfection, fluorescent-positive cells were sorted, and DNA was extracted for downstream DNA methylation analysis.

### Viral particle transduction and triple-fluorescent-positive cell purification

We added 4 μg/ml polybrene (Sigma) to 10% FBS and 1% P/S DMEM. HEK293T cells were transduced with lentiviral particles of dCas9^SunTag^, ^scFv^DNMT3A1, and sgRNA-RFP657 in the polybrene-containing media. Spin transduction was conducted at 1100 rpm for 2 h at room temperature. The transduced cells were moved to an incubator overnight. After 48 h, fluorescence-positive cells were purified by flow cytometry (BD FASCAria II). Fluorescence-positive cells were placed in changed media every 2–3 days. After 30 days, fluorescence-positive cells were repurified for the subsequent methylation and gene expression analysis.

### Analysis of DNA methylation in targeted regions

HEK293T cells, either transfected or transduced with dCas9-SunTag-DNMT3A vectors, were isolated using flow cytometry. The genomic DNA of the isolated cells was extracted using a PureLink Mini Kit (Invitrogen) and then bisulfite converted using an EpiTect Bisulfite Kit (Qiagen) for the following deep sequencing. Bisulfite PCR primers are listed in Additional file 2: Table S1. Ten microliters of Zymotaq DNA polymerase premix, 1 μl for each forward and reverse primer, 6 μl double-distilled water (ddH_2_O), and 2 μl bisulfite-treated DNA were mixed and run according to the following PCR program. First, samples were heat activated at 95 °C for 5 min; for the second step, they were kept at 95 °C for 30 s, then at 60 °C for 2 min and 30 s, and then decreased 0.2 °C every cycle, 72 °C for 2 min and 30 s. Then the process was repeated from the second step for 40 cycles, and finally extended at 72 °C for 10 min. Bisulfite PCR products were run in 2% agarose electrophoresis, excised, and extracted using a gel extraction kit (Qiagen). The DNA concentration of gel extracted products was measured using a Qubit dsDNA HS Assay Kit (Life Technologies) and adjusted to 0.2 ng/μl for Nextera libraries preparation. The Nextera libraries preparation was based on the manufacturer’s instructions (Illumina). The paired-end sequencing reads were aligned to the human genome (hg19) by the software Bisulfite Sequence MAPping (BSMAP) 2.90 [27], and low-quality sequences were trimmed as the default threshold. We computed the methylation ratios of CpGs and CpHs with coverage depth at least 1000× using the BSratio tool of BSMAP.

### Quantitative PCR

RNA was extracted from purified triple-fluorescent-positive cells using an RNeasy Micro Kit (Qiagen). We mixed 1 μg RNA of each sample with 1 μl oligo dTs and 1 μl 10 mM deoxynucleotide triphosphates (dNTPs) and ddH_2_O. RNA mixtures were heated to 65 °C for 5 min and incubated on ice for 1 min. We then added 4 μl 5X First-strand buffer, 1 μl 0.1 M dithiothreitol (DTT), 1 μl RNase inhibitor, and 1 μl SuperscriptIII Reverse Transcriptase (RT) to the RNA mixture, incubated it at 50 °C for 60 min, and inactivated the reaction by heating to 70 °C for 15 min. Then 0.5 μl cDNAs, 0.5 μl of 10 μM forward and reverse primers, 3.5 μl ddH_2_O, and 5 μl 2X SsoAdvanced Universal SYBR Green Supermix were added to PCR tubes and qPCR was conducted as follows. First samples were heat activated at 95 °C for 3 min, then secondly kept at 95 °C for 10 s, then at 55 °C for 10 s, and at 72 °C for 30 s. The process was repeated from the second step for 40 cycles. The qPCR primers are listed in Additional file 2: Table S1.

### Reduced representation bisulfite sequencing and analysis

The RRBS was conducted as previously described [28]. Briefly, genomic DNA was diluted to 20 ng/μl in low TE buffer (10 mM Tris-HCl, 0.1 mM ethylenediaminetetraacetic acid (EDTA), pH = 8.0). Three hundred nanograms of genomic DNA was digested in 1X NEB buffer 2.1 and *Msp*I at 37 °C overnight. *Msp*I-digested DNA was added with Klenow fragments and a dNTPs mixture (10 mM dATPs, 1 mM dCTPs, and 1 mM dGTPs) and incubated at 30 °C for 20 min and then at 37 °C for 20 min. A 2X volume of AMPure XP beads was added to each sample, and the samples were incubated at room temperature for 30 min. The beads were placed on a magnetic stand for 5 min, the supernatants were removed, and the beads were washed twice with 70% ethanol. After the beads were air-dried for 15 min, the DNA was eluted with EB buffer and ligated with 10X diluted Illumina TruSeq adapters at 16 °C overnight. After the ligases were inactivated at 65 °C for 20 min, a 2X volume of AMPure beads was added to the DNA and incubated for 30 min. The beads were washed as previously described, and the DNA was eluted in EB buffer and bisulfite converted using an EpiTect Bisulfite Kit (Qiagen). The bisulfite-treated DNA was amplified according to the following program. First samples were heat activated at 98 °C for 30 s, then secondly kept at 98 °C for 10 s, then at 60 °C for 30 s, and 72 °C for 30 s. The process was repeated from the second step for 22 cycles. The PCR-amplified DNA was purified using AMPure beads as described. Libraries were sequenced using a NextSeq 500 sequencer.

Reads of four libraries including Neg, ^scFv^DNMT3A1, dCas9^SunTag^ + ^scFv^DNMT3A, and dCas9^SunTag^ + ^scFv^DNMT3A1 + sgHOXA5 were mapped to the human genome (hg19) using BSMAP 2.90 [27], and low-quality sequences were trimmed as the default threshold. The methylation ratios of CpGs (sequencing depth ≥ 5) were computed with the BSratio tool of BSMAP. The covering regions of RRBS are enriched in CpG-rich regions such as CpG islands (CGIs). Human CGIs (hg19) were downloaded from the UCSC Browser Table. We examined the 5982 CGIs commonly covered by all of four RRBS library fragments (Additional file 3: Table S2). Among these common CGIs, the differentially methylated CGIs were identified by an absolute methylation difference > 0.2 between the paired samples and *P* values < 0.01 adjusted by the Benjamini-Hochberg method using one-way analysis of variance (ANOVA).

### RNA sequencing and analysis

RNA from dCas9-SunTag-DNMT3A-treated samples was extracted using an RNeasy Micro Kit (Qiagen) and quantified using Nanodrop. The TruSeq stranded mRNA library preparation was based on the manufacturer’s instructions (Illumina). Libraries were sequencing using a NextSeq 500 sequencer. Paired-end RNA-seq reads were mapped to the human genome (hg19) using TopHat 2.0.10. The fragments per kilobase of exon per million fragments mapped (FPKM) values were calculated using Cufflinks 2.2.1.

### Whole genome bisulfite sequencing (WGBS) and analysis

One hundred nanograms of DNA was used for WGBS libraries preparation using a TruSeq DNA methylation kit according to the manufacturer’s instructions (Illumina). Libraries were sequenced using a NextSeq 500 sequencer. For each WGBS profile, we used BSMAP to trim adapter and low-quality sequences as the default threshold and aligned the bisulfite-treated reads to the human genome (hg19). Then the methylation ratio of each CpG covered with at least five reads was calculated by the module BSratio in BSMAP.

## Additional files

Additional file 1: Figures S1–S11. Supplementary (PDF 3683 kb)

Additional file 2: Table S1. List of primers used in this study. (XLSX 17 kb)

Additional file 3: Table S2. Differentially methylated regions identified in RRBS. (XLSX 630 kb)
