# Supplementary material for: DNA epigenome editing using CRISPR-Cas SunTag-directed DNMT3A
Source: Genome Biol. 2017 Sep 18;18:176. doi: 10.1186/s13059-017-1306-z (PMC5604343; doi:10.1186/s13059-017-1306-z)
Supplement: Supplementary file 1 — Supplementary (PDF 3683 kb) [file 13059_2017_1306_MOESM1_ESM.pdf]

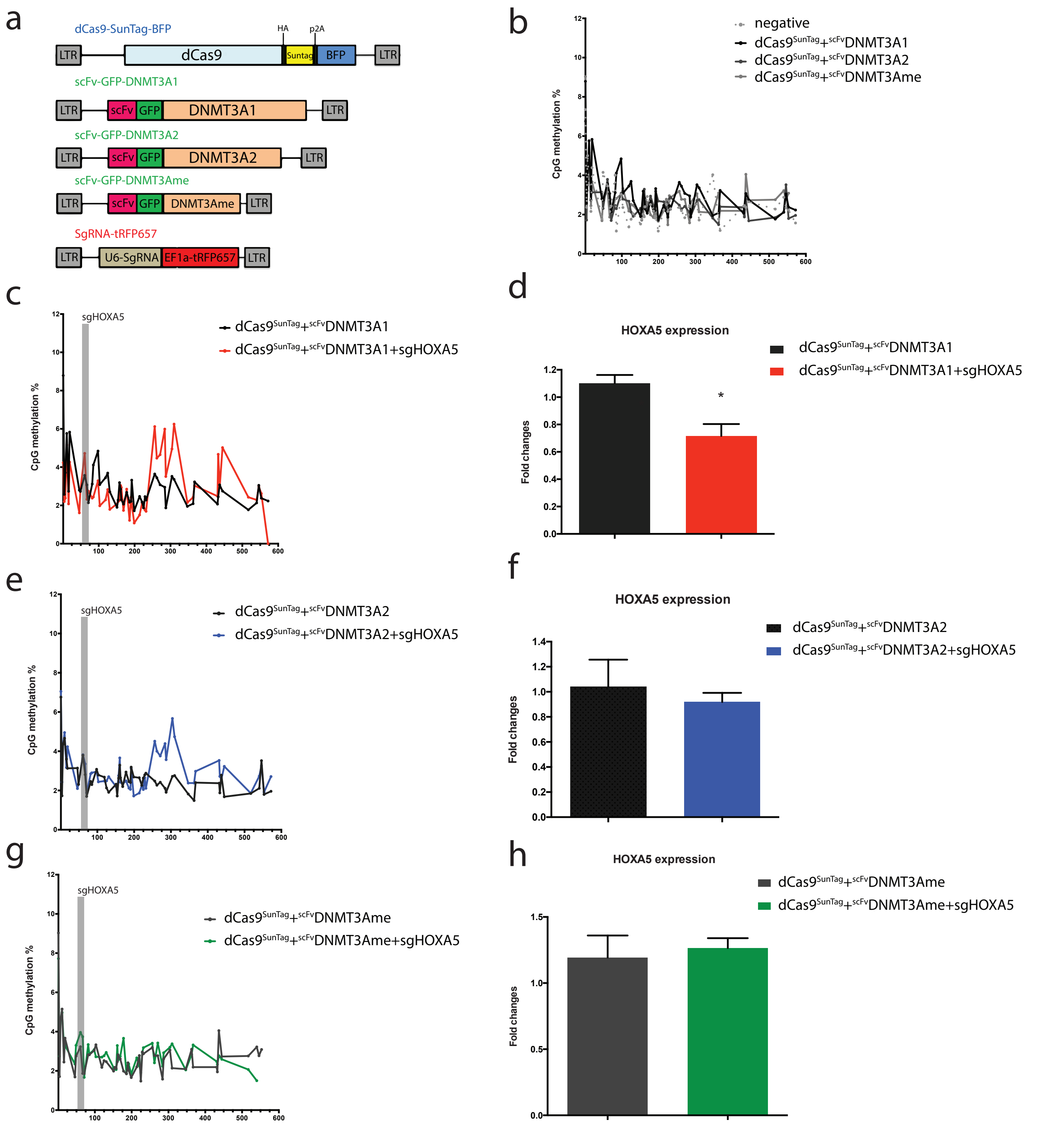

Figure S1: Transient transfected dCas9-SunTag-DNMT3A1 increased 2~5% CpG methylation at the *HOXA5* locus and repressed 20% HOXA5 expression. (a) Schematic of lentiviral vectors of dCas9-SunTag-DNMT3A system. (b), (c), (e), (g) The graph depicts percent of methylation across individual CpGs as determined by deep sequencing of amplicons at the *HOXA5* locus. (d), (f),(h) The graph depicts fold-changes of HOXA5 expression as analyzed by quantitative real-time PCR for HOXA5 expression. Significance was calcualted by student t test. * $p<0.05$.

Huang, YH et al.

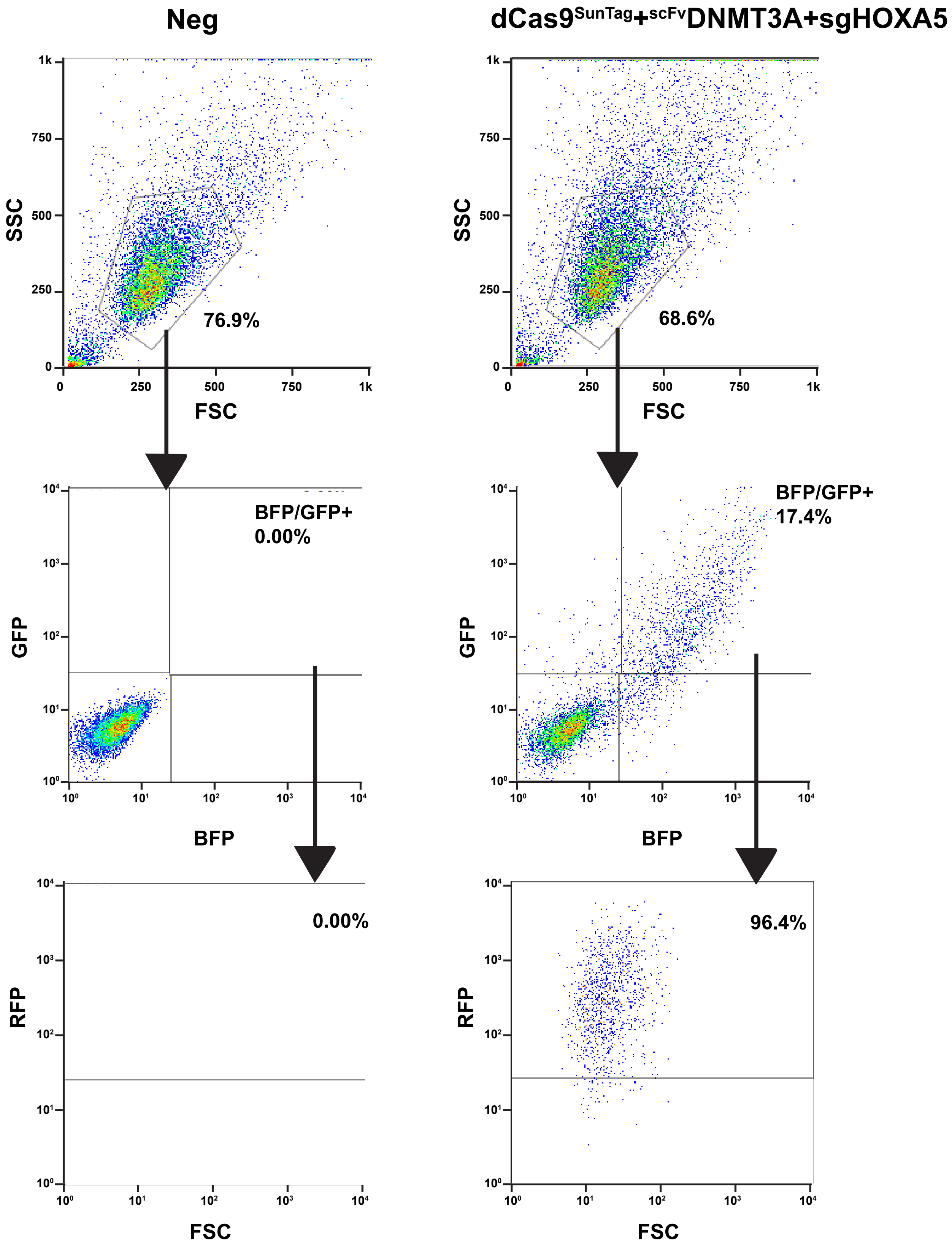

Figure S2: Demonstration of sorting scheme using flow cytometry for dCas9-SunTag-DNMT3A system. The graph depicts the percent of fluorescence-positvie cells in untranfected and transfected dCas9-SunTag-DNMT3A1 cells as determined by flow cytometry. Singlet HEK 293T cells were gated by FSC and SSC and then blue, green fluorescence-positive cells were gated. Triple fluorescence-positive cells were gated from BFP/GFP double positive cells.

Huang, YH et al.

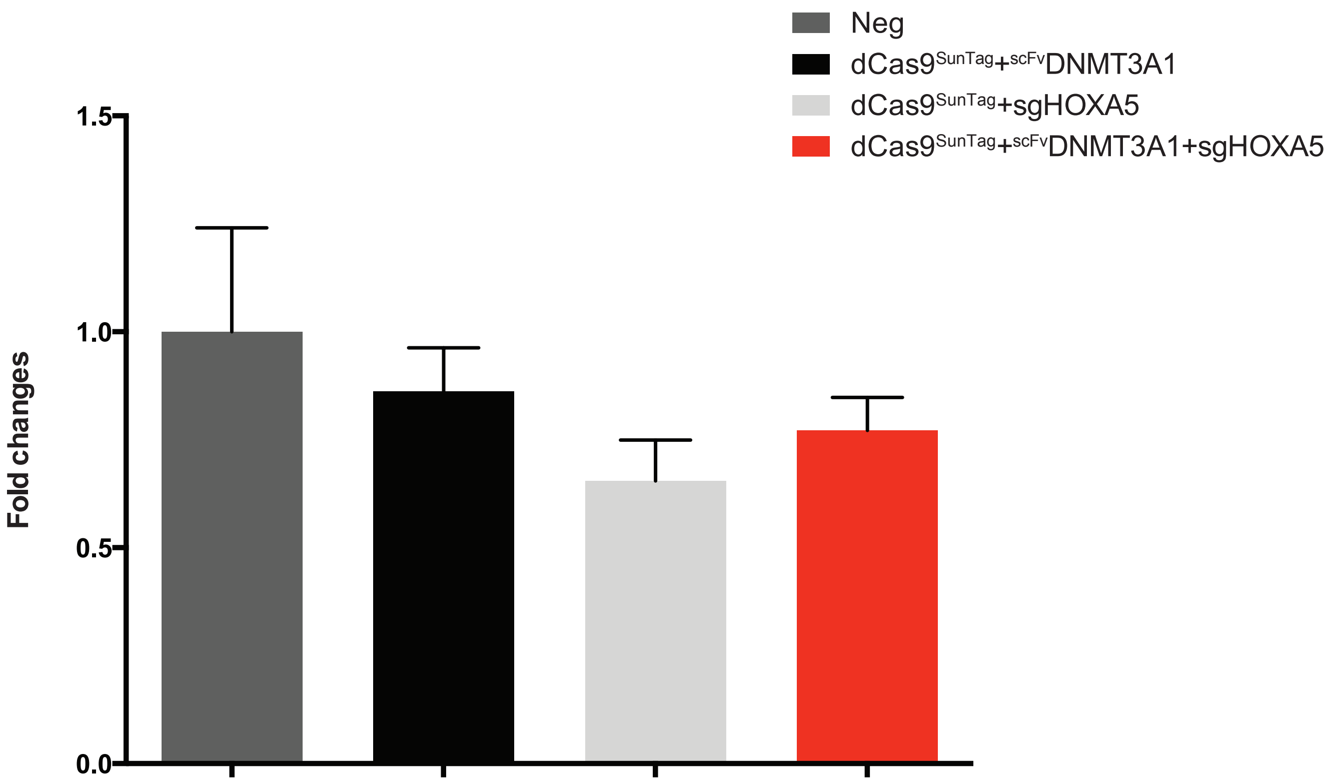

Figure S3: dCas9 binding affects transcription expression of HOXA5.
The graph depicts the fold-change relative to the untransfected sample analyzed by quantitative real-time PCR for HOXA5 expression, measured two days after transfection of indicated constructs.

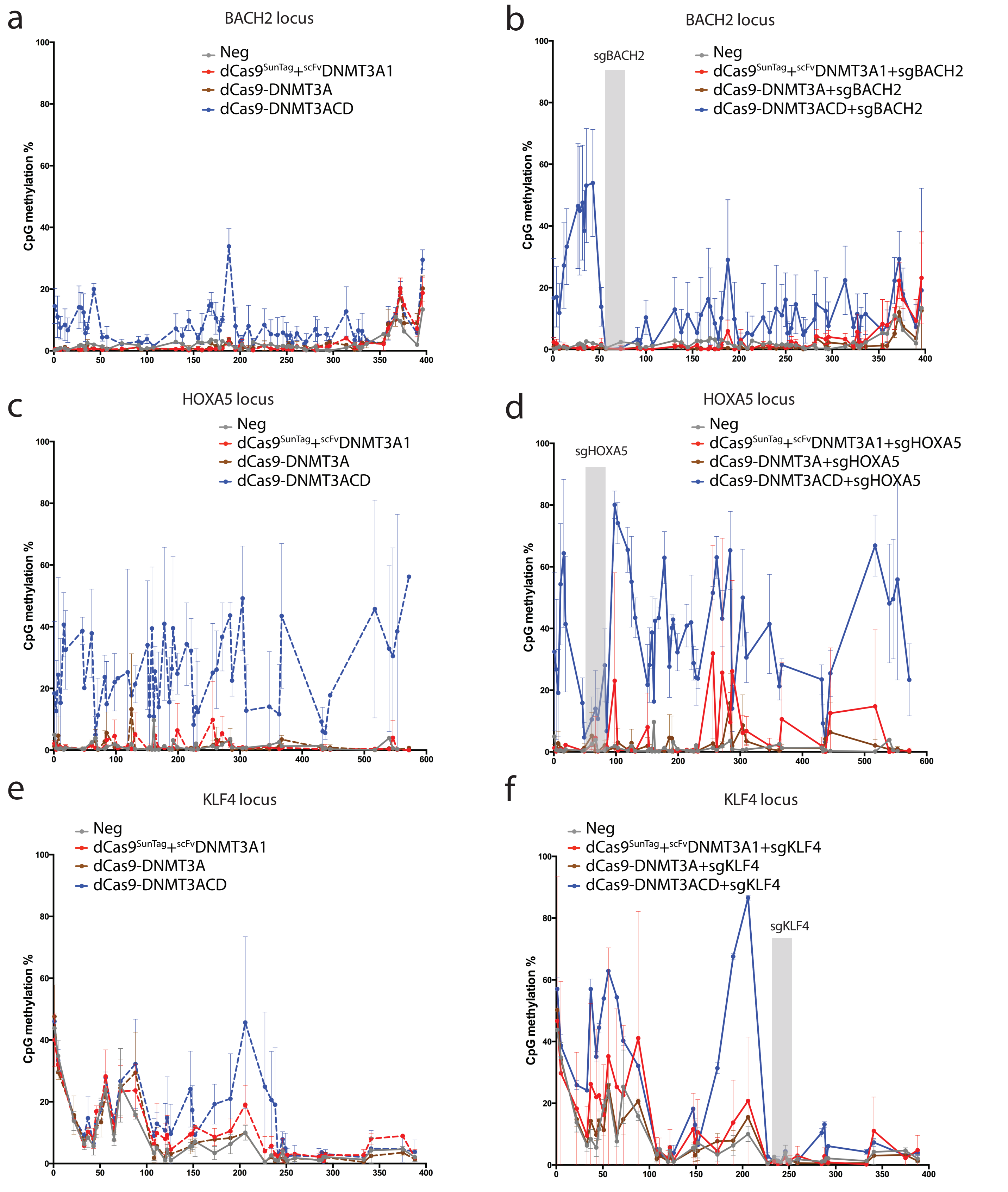

Figure S4: Direct Comparison of dCas9-SunTag-DNMT3A system with the Existing tools. (a),(b) The graph depicts percent of methylation across individual CpGs of three biological replicates as determined by deep sequencing of amplicons at the *BACH2* locus. (c),(d) The graph depicts percent of methylation across individual CpGs of three biological replicates as determined by deep sequencing of amplicons at the *HOXA5* locus. (e), (f) The graph depicts percent of methylation across individual CpGs of three biological replicates as determined by deep sequencing of amplicons at the KLF4 locus.

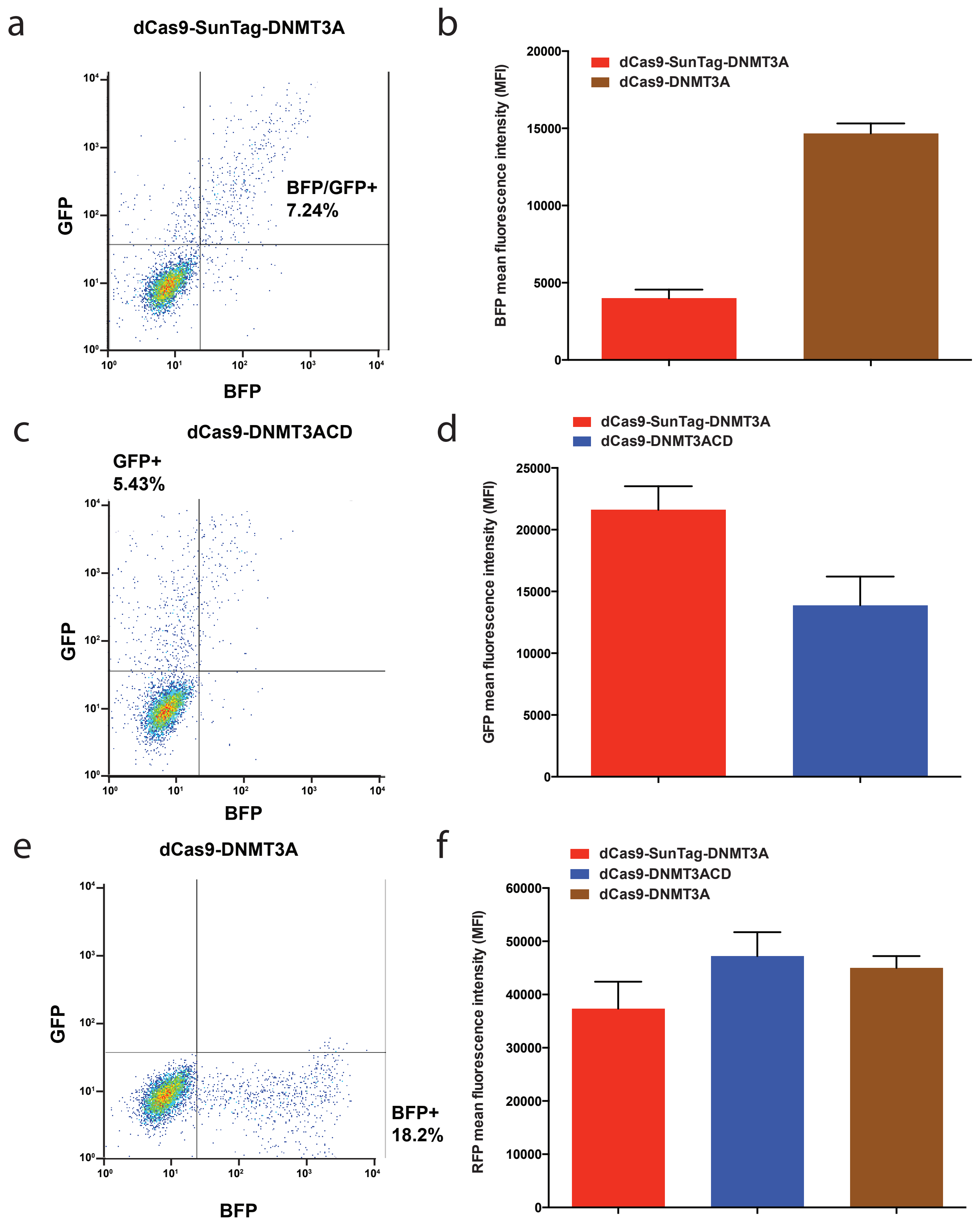

Figure S5: Expression level of the components in direct comparison.
(a),(c),(e) The graph depicts the percent of fluorescence-positvie cells in direct comparison experiments as determined by flow cytometry. (b),(d),(f) The graph depicts mean fluorescence intensity of BFP, GFP and RFP in the direct comparison experiments. (n=5)

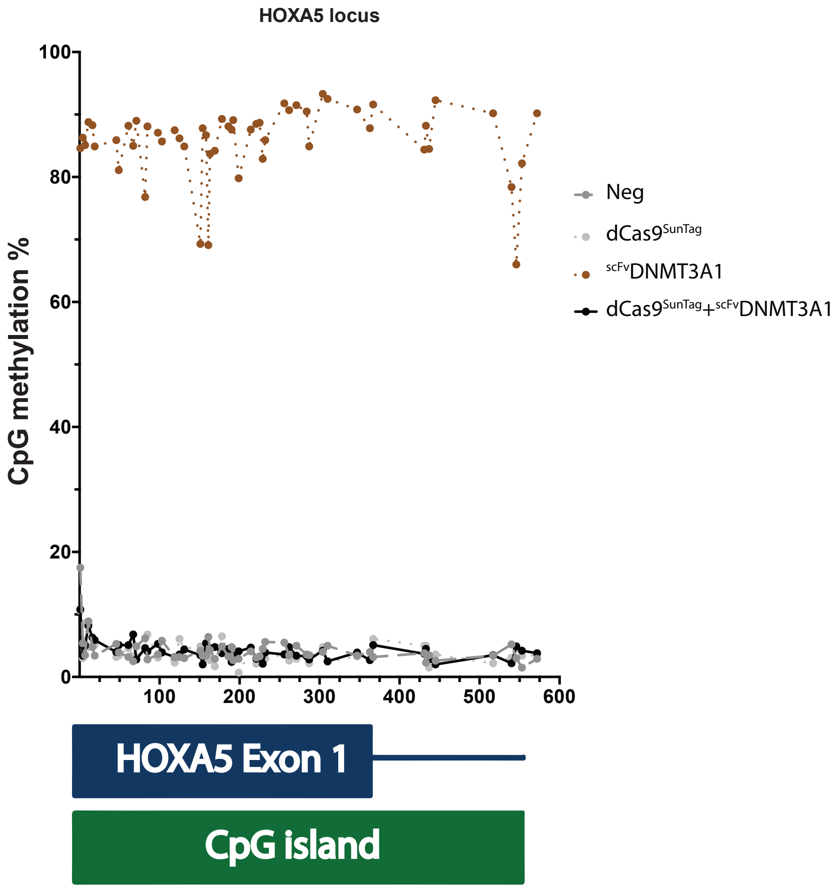

Figure S6:
The graph depicts percent of methylation across individual CpGs as determined using deep sequencing of amplicons at the *HOXA5* locus.

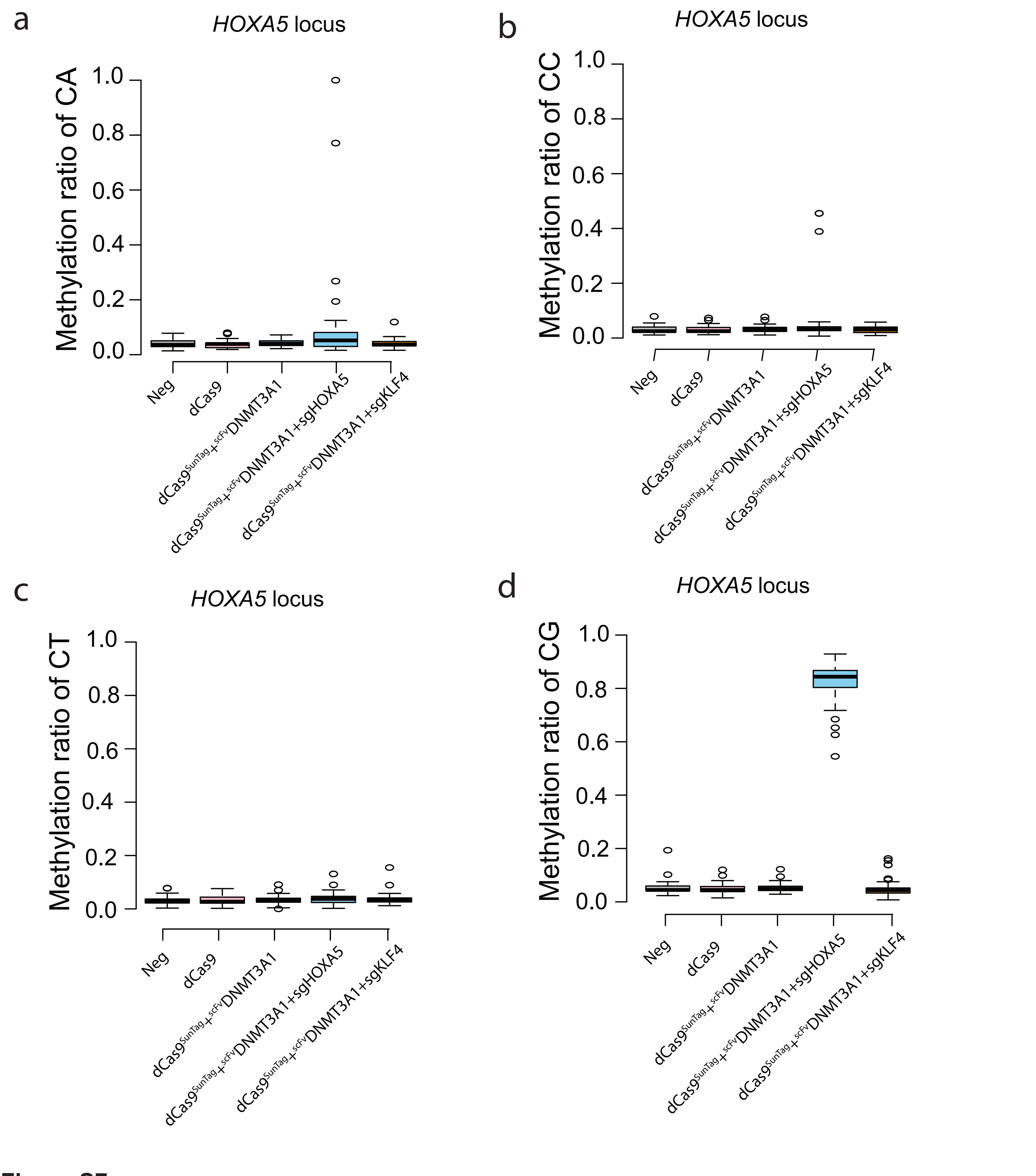

Figure S7:
The graphs depict methylation ratio of CA (a), CC (b), CT (c) and CG (d) as ploted by boxplot and determined using deep sequencing of amplicons at the *HOXA5* locus. Box plot represents the interquartile range (25–75%) with median. Whiskers represents 2.5 and 97.5 percent.

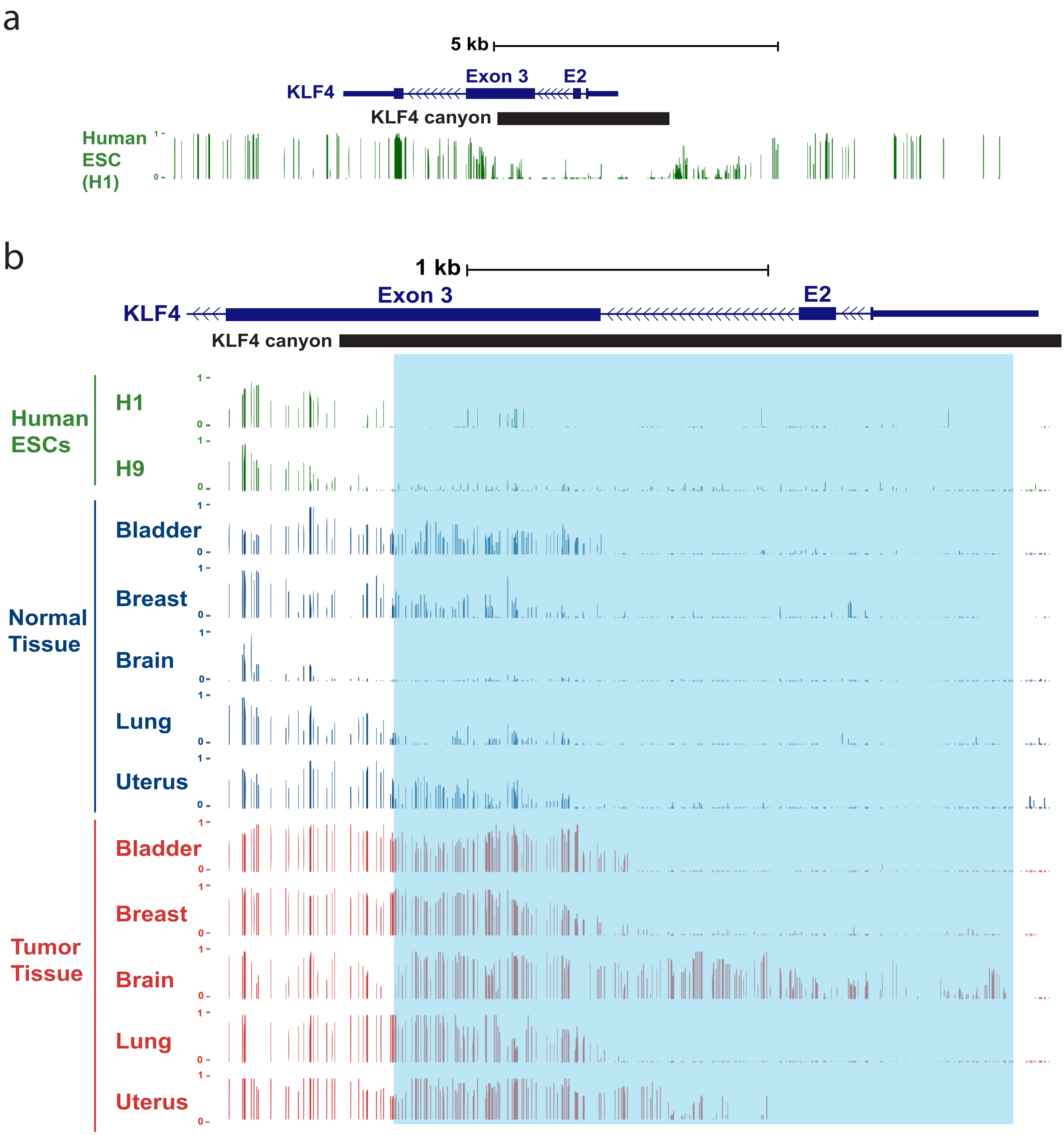

Figure S8:
(a) The graph depicts level of methylation across individual CpGs at the canyon of *KLF4* locus in human embryonic stem cells (H1) as determined using whole genome bisulfite sequencing (WGBS). 0 on the scale represents 0% methylation at each CpG. 1 represents 100% methylation. (b) The graph depicts level of methylation across each CpG at the *KLF4* locus in different types of cells and tumor samples as determined using WGBS. The blue shaded area corresponds to transition regions, which became highly methylated in many types of tumor tissues.

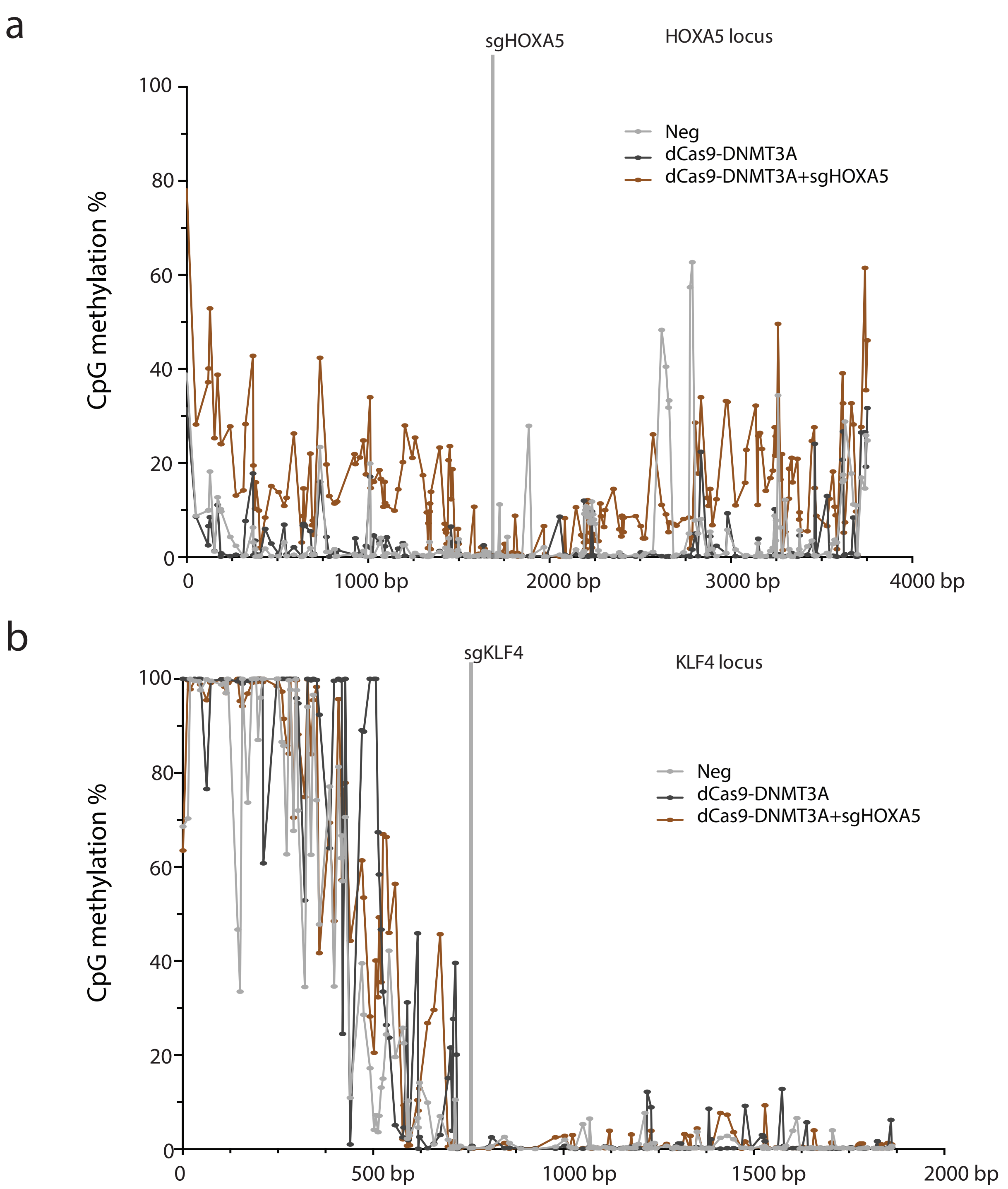

Figure S9: dCas9-DNMT3A system showed 20-40% increased DNA methylation after 30 days incubation. (a)The graph depicts percent of methylation across individual CpGs as determined by deep sequencing of multiple amplicons at the *HOXA5* locus. (b) The graph depicts percent of methylation across individual CpGs as determined by deep sequencing of multiple amplicons at the *KLF4* locus.

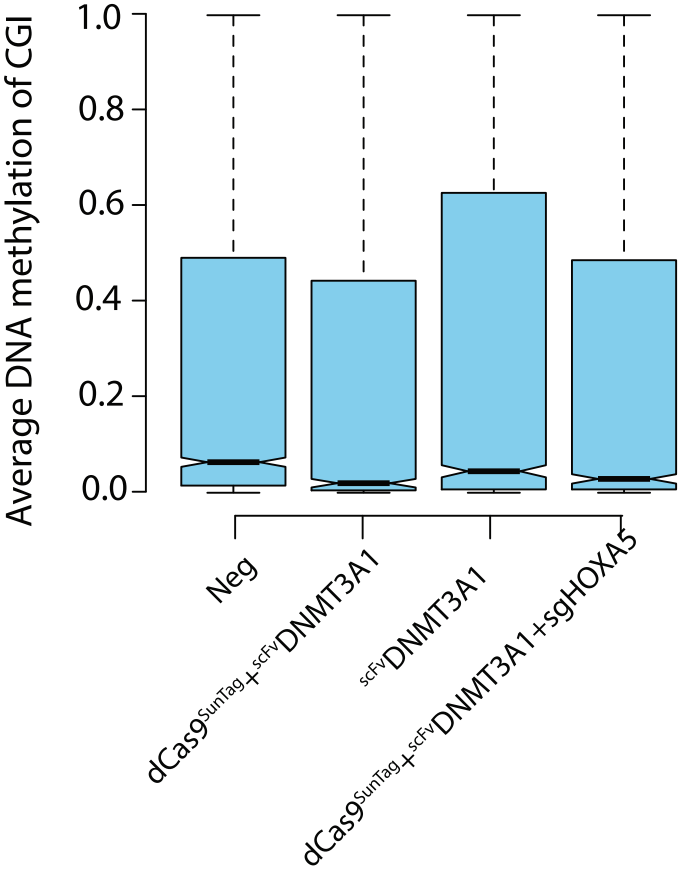

Figure S10:
The graph depict distributions of average methylation level of CpGs in the CpG islands (CGI) as determined by reduced representation bisulfite sequencing and ploted using box plot. Box plot represents the interquartile range (25–75%) with median. Whiskers represents 2.5 and 97.5 percent.

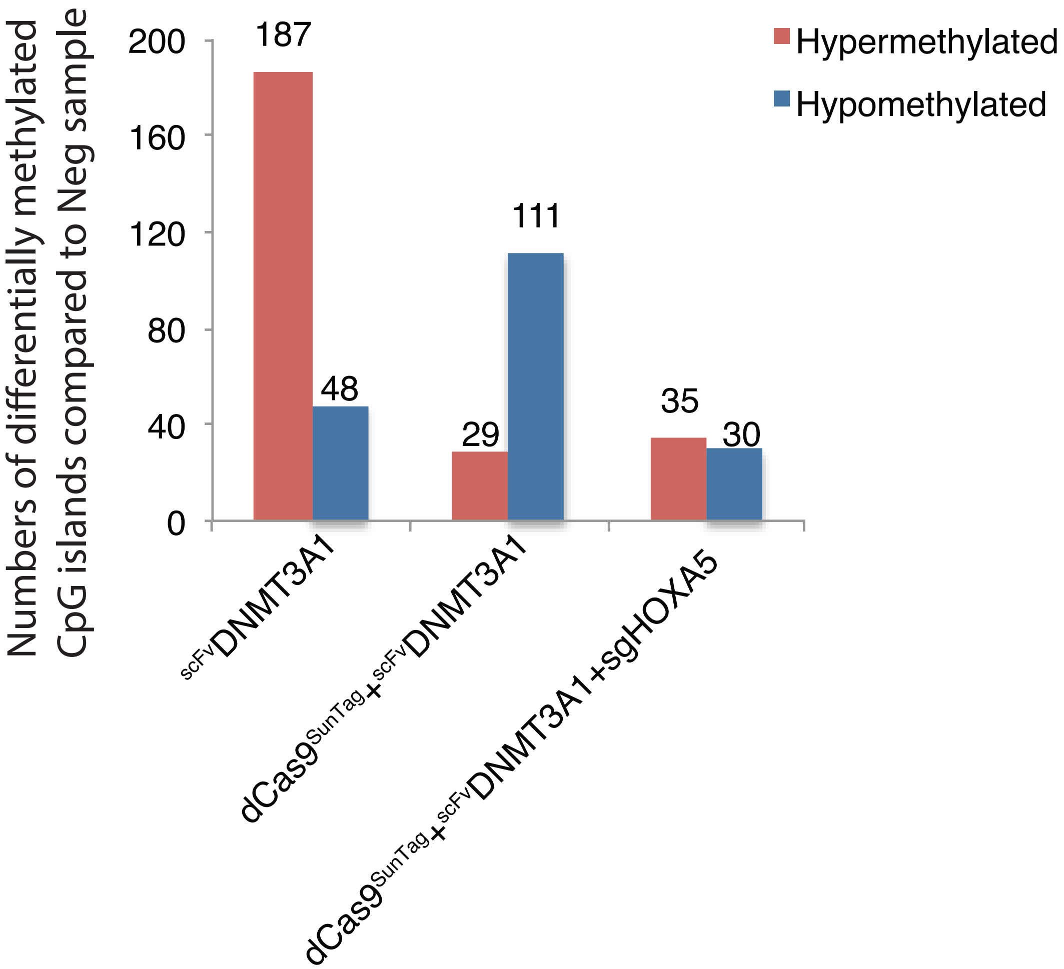

Figure S11: Quantitative analysis of reduced representation bisulfite sequencing (RRBS). The graph depicts numbers of differentially methylated CpG islands (CGIs) compared to Neg sample within 5982 commonly identified CGIs as determined by RRBS. The differentially methylated CGIs were identified by the absolute methylation difference >0.2 between the paired samples and p-values <0.01 adjusted by Benjamini Hochberg method using one-way ANOVA analysis.
